# University students’ experiences regarding elder abuse: Grounded Theory

**DOI:** 10.1590/0034-7167-2025-0290

**Published:** 2026-01-09

**Authors:** Juliana Ribeiro da Silva Vernasque, Maria Clara de Sousa Santos, Ana Laura Lopes Loosli, Larissa Rodrigues, Paula Sales Rodrigues, Fabiana Veronez Martelato Gimenez, Miriam Fernanda Sanches Alarcon, Maria José Sanches Marin

**Affiliations:** I Faculdade de Medicina de Marília. Marília, São Paulo, Brazil; II Universidade Estadual do Norte do Paraná. Bandeirantes, Paraná, Brazil

**Keywords:** Elder Abuse, Violence, Universities, Students, Grounded Theory., Abuso de Ancianos, Violencia, Universidades, Estudiantes, Teoría Fundamentada.

## Abstract

**Objectives:**

to interpret young university students’ experiences regarding elder abuse and develop a theoretical model.

**Methods:**

a qualitative study, based on Strauss’ approach of Grounded Theory and Bauman’s theoretical framework, with theoretical saturation sampling, in which 34 university students from different areas were interviewed. Data processing used NVivo software. The study was approved by the Research Ethics Committee.

**Results:**

participants highlighted different types of elder abuse as well as their invisibility and trivialization in society. Older adults’ frailty and vulnerability were identified as risk factors, suggesting preventive strategies, including the inclusion of the topic in academic curricula, the promotion of healthy aging, and the strengthening of public policies.

**Final Considerations:**

the theoretical model developed highlights the urgent need for educational interventions based on a critical understanding of this problem, with an emphasis on human sensitivity and the morality of social interactions.

## RESUMEN

**Objetivos:**

interpretar las experiencias de jóvenes universitarios respecto a la violencia contra ancianos y desarrollar un modelo teórico.

**Métodos:**

estudio cualitativo, basado en el enfoque straussiano de la Teoría Fundamentada y el marco teórico de Bauman, con muestreo de saturación teórica, en el que se entrevistó a 34 estudiantes universitarios de diferentes áreas. El procesamiento de datos se realizó mediante el software NVivo. El estudio fue aprobado por el Comité de Ética de la Investigación.

**Resultados:**

los participantes destacaron las diferentes formas de violencia contra los ancianos, así como su invisibilidad y trivialización en la sociedad. La fragilidad y la vulnerabilidad de los ancianos se identificaron como factores de riesgo, sugiriendo estrategias preventivas, como la inclusión del tema en los currículos académicos, la promoción del envejecimiento saludable y el fortalecimiento de las políticas públicas.

**Consideraciones Finales:**

el modelo teórico desarrollado destaca la urgente necesidad de intervenciones educativas basadas en una comprensión crítica de este problema, con énfasis en la sensibilidad humana y la moralidad de las interacciones sociales.

## INTRODUCTION

The current demographic and epidemiological transition resulting from population aging requires significant societal efforts, as older adults have complex and chronic needs, including managing long-term health conditions, ensuring access to adequate healthcare, and addressing the social, emotional, and economic challenges associated with aging. This issue is particularly pressing in less developed countries, where the rapid pace of population aging exacerbates existing inadequacies, such as insufficient health infrastructure and policies to support older adults, as well as a lack of public awareness of the complexities of aging^(1)^.

The aging process involves morphological, functional, biochemical, and psychological changes^(2)^ that can result in loss of functional capacity, the emergence of diseases, geriatric syndromes, hospitalizations, and social, family, and intergenerational conflicts. Consequently, older adults experience a loss of autonomy and independence, increasing their exposure to risk factors, including violence^(3)^.

The global growth of the population over 60 has resulted in a high prevalence of elder abuse, highlighting the need for new strategies to address this issue. This human rights violation involves any harmful action or omission in a relationship of trust^(4)^.

Elder abuse is a complex and preventable global phenomenon that can lead to psychological or physical distress, deprivation of liberty, or even death. It manifests itself in many types, including neglect, self-inflicted violence, physical abuse, sexual violence, financial exploitation, abandonment, and psychological or emotional abuse. It currently represents a significant problem for society, particularly for public health, requiring intersectoral action. However, many professionals report limited intervention capabilities, along with fears of reporting and uncertainty about the roles of other services, further complicating the response to elder abuse^(3)^.

As an interdisciplinary issue, elder abuse encompasses the humanities, exact sciences, and biological sciences. However, it is often underfunded, underexamined, and underrecognized, receiving inadequate attention from professionals in all fields^(5)^.

In this context, the training of future professionals, from all areas of knowledge, can play a fundamental role in identifying and preventing elder abuse, preparing university students to become agents of change^(6)^.

Universities, through their role in teaching, research and providing services to the community, play an important role in disseminating values and beliefs that lead to the development of daily actions that can positively influence society’s worldview, creating a culture focused on humanization precepts, especially when it comes to a society in a rapid aging process and with specific demands^(7)^.

It should be added that universities can lead the training of conscious professionals with a comprehensive vision of sustainability, inclusive economy and social justice, and this approach must be integrated into the school curriculum in a transversal manner in all areas of knowledge, through active pedagogical practices that aim at critical reflection on reality and its transformation^(8)^.

It is therefore clear that university students represent a segment of future professionals who will directly engage with older populations and are important agents of change in their various contexts. However, studies indicate that the topic of elder abuse is rarely systematically addressed in undergraduate curricula, limiting these future professionals’ preparedness to intervene in situations of violence^(9)^.

Given this context, the question arose: how do university students from different fields of study experience elder abuse?

## OBJECTIVES

To interpret young university students’ experiences regarding elder abuse and develop a theoretical model.

## METHODS

### Ethical aspects

The research was approved by the Research Ethics Committee, following the Brazilian National Health Council ethical precepts. Interviews were conducted using Google Meet^®^, and participants signed an Informed Consent Form after understanding and agreeing. To maintain anonymity, interviewees were identified by the letter “I”, followed by the interview order number and the initials of their field of study, such as social sciences (SOCSCI), law (LAW), nursing (NUR), civil engineering (CIVENG), electrical engineering (ELEENG), medicine (MED), nutrition (NUT), pedagogy (PED), and occupational therapy (OT).

### Study design

This is qualitative research that used Grounded Theory (GT), in Strauss’ perspective, which is constructed through the interaction between the researcher and the data itself^(10)^. Strauss’ approach was chosen for its ability to explore dynamic social processes and unravel the complex interactions between social, cultural, and institutional factors underlying elder abuse. This methodological approach allows for greater researcher involvement in the interpretation and modeling of emerging theories, ensuring a comprehensive understanding of the phenomenon^(10)^.

To maintain methodological rigor, the COnsolidated criteria for REporting Qualitative research protocol was used as a support tool for research development.

### Theoretical framework

The theoretical model is based on Zygmunt Bauman’s work, who recognizes violence as part of a complex process in humanity. Bauman posits that without intervention to break the cycles of violence, today’s victims may become tomorrow’s aggressors^(11-13)^.

Furthermore, contemporary society is characterized by the “liquidity” of social relations, in which support networks’ fragility and interpersonal interactions’ inconsistency directly affect individuals’ vulnerability, particularly of older adults^(11)^. This fragility of human and social relationships contributes to marginalization of older adults and perpetuation of abuse, presenting a significant challenge for future generations of professionals.

### Study setting

The study was carried out in a medium-sized municipality in the Central-West region of the state of São Paulo, with an estimated population of 237,627 inhabitants in 2022, of which 44,835 were aged 60 or over^(14)^. The municipality boasts a large complex of Higher Education Institutions (HEIs), encompassing both public and private institutions offering programs in various fields. For this study, four HEIs were selected for convenience, two public and two private. The objective was to include health sciences, exact sciences, and humanities university students, creating a diverse sample composition, as suggested by the GT, and to obtain theoretical variation and analytical depth.

### Sample

The sample was defined by theoretical saturation, which was considered fully achieved after the thirty-fourth interview, conducted with humanities, exact sciences, and health sciences university students from two public and two private institutions. Inclusion criteria were being enrolled in one of the four participating institutions, having completed at least one year of study, and being 18 years of age or older. Exclusion criteria included being unavailable to participate in the interview or having transferred from another institution less than six months prior.

### Data collection

Semi-structured interviews served as the primary data collection method, appropriate for investigating social phenomena and capturing individual perceptions^(15)^. The interviews, conducted by a doctoral student experienced in interview techniques, took place from January 2022 to September 2023. This period allowed each interview to be transcribed and analyzed sequentially in a continuous process.

Using the script, sociodemographic information (age, sex, marital status, year of study, and field of study) was collected, including the following guiding questions: what do you know about elder abuse that you can share with us? How do you perceive elder abuse in society? How important is it to address this topic during undergraduate studies? Do you have suggestions for improving the approach to this topic? What does this topic mean to you personally? How would you respond to a situation involving elder abuse?

Each interview lasted approximately 30 minutes and was transcribed and analyzed immediately afterward. They were conducted with a focus on the subject of study, and the simultaneous transcription and analysis allowed for minor adjustments to the questions as needed, ensuring a deeper understanding of the topic studied.

### Data analysis

The analysis was conducted according to GT principles, from Strauss’ perspective, involving the “open coding”, “axial coding”, and “selective coding” stages^(15)^. NVivo 14 software was used to facilitate qualitative data organization and systematization, ensuring traceability, consistency, and reliability of the analytical process. Coding was performed by the first author and verified by the last author to ensure accuracy and reliability. MindMeister software was used to illustrate the theoretical model.

The coding process followed a continuous path, conceptualizing data according to characteristics, comparing and grouping concepts, which generated preliminary categories and, finally, the central category.

Initially, the data were subjected to line-by-line microanalysis to identify meaningful codes derived directly from the interview transcripts. This stage resulted in 577 codes. For instance, when reporting reading a short story by Clarice Lispector about the abandonment of an older adult, one student stated: *“When you talk about elder abuse, the first thing that comes to mind is a story by Clarice* [...] *it always had a big impact, one scene that stuck with me is her covered in breadcrumbs”.* From this excerpt, actions such as “naming the story”, “relating to abandonment”, and “expressing emotional impact” were coded, highlighting how literary narratives influence older adults’ perceptions of violence and abandonment.

Subsequently, in open coding, the codes were grouped into concepts or elements that shared thematic similarities, producing 50 concepts. For instance, the codes “*Understanding neglect as elder abuse*” (I1CIVENG) and “*Considering that the older population is neglected and that violence occurs frequently within the family and in institutions*” (I2NUR) were grouped under the concept/element “neglect”.

Axial coding involved establishing relationships between the 50 concepts/elements to formulate the 14 preliminary categories/subcategories, enabling a deeper understanding of the dynamics underlying elder abuse based on university students’ experiences.

The final stage, selective coding, was refined into four categories, integrating the 14 subcategories based on shared properties and dimensions to form the central category, which allowed synthesizing the interconnected processes of students’ experiences related to elder abuse.

The model’s validity was strengthened through peer reviews by experienced GT researchers and external validation in two remote meetings, which included the participation of five student interviewees, two GT experts, and three members of the research group, confirming the representativeness and interpretative fidelity of the proposed theoretical model.

## RESULTS

Thirty-four university students from different fields, programs, and HEIs participated in the study. Twenty-two (64.7%) identified as women and 12 (35.3%) as men. Their ages ranged from 19 to 44, with a mean of 24.47, revealing a predominantly young group, but with varied age ranges.

The sample comprised students from nine different programs, distributed between the 2^nd^ and 5^th^ years of undergraduate studies, as detailed in Chart 1. This diversity contributed to broadening the scope of perceptions and reflections on the topic under investigation. Concerning their institution of origin, 18 participants were enrolled in public universities and 16 in private institutions, reinforcing the study’s commitment to institutional representation.

**Chart 1:** Participant characteristics by course and year of graduation, Marília, São Paulo, Brazil, 2025

Undergraduate course/year	SOCSCI	LAW	NUR	CIV ENG	ELE ENG	MED	NUT	PED	OT	Overall total
2^nd^ year	2	4		1				2		9
3^rd^ year	1			1	1		3	1	1	8
4^th^ year			4	1		4	1		3	13
5^th^ year				4						4
**Overall total**	**3**	**4**	**4**	**7**	**1**	**4**	**4**	**3**	**4**	**34**

*SOCSCI - social sciences; LAW - law; NUR - nursing; CIVENG - civil engineering; ELEENG - electrical engineering; MED - medicine; NUT - nutrition; PED - pedagogy; OT - occupational therapy.*

The coding process revealed as a central phenomenon “Acknowledging the complexity of elder abuse and the need for effective interventions”, which was evidenced by the categories “Identifying different types of violence”, “Acknowledging older adults’ needs”, “Considering the trivialization of elder abuse” and “Proposing actions to reduce elder abuse”, as presented below.

### Identifying different types of violence

In this category, interviewees recognized different types of violence, occurring both at home and in institutions, perpetrated mainly by family members.

For participants, the types in which elder abuse manifests itself include physical violence, financial violence, psychological violence, sexual violence, neglect, omission, disrespect, ageism, and denial of dignity, which translates into acts of deprivation, abandonment, isolation, impatience, and insults, as observed in the following statements.

*Because it’s not just physical violence. What we see is financial abuse, which older adults suffer from family members who take advantage of them, emotional abuse, psychological and sexual violence as well* [...]. (I2TO)

*Elder abuse is often not obvious. It can manifest itself in a failure to help an older adult.* (I4LAW)

*Interfering with their dignity, wanting to belittle them because of their age, not giving them the attention they need.* (I4NUT)

These types of violence generally occur in domestic settings, primarily perpetrated by family members. Furthermore, participants highlighted that institutional settings, such as healthcare services and Nursing Homes (NHs), also present risks of neglect, abandonment, and prolonged isolation.

*There was an older adult woman who lived alone and was mistreated by her children. They extorted all the money she received from child support and only visited her to collect the money, neglecting her basic needs.* (I1MED)

*In my city, there’s a nursing home with many older adults who don’t receive visitors. Most of them seem visibly sad due to the lack of family presence.* (I4LAW)

University students demonstrated awareness of the problem that elder abuse represents, as they had already experienced this type of situation with family members or close people, learned about it through the media, or heard about it from other people.

[...] *ah, I have personal experiences, from my family and experiences that I had in my course, like that.* (I4MED)

[...] *I’ve heard cases, mostly in the media, that this issue of elder abuse is quite prevalent.* (I4NUR)

### Acknowledging older adults’ needs

Participants emphasized older adults’ specific needs, which occur due to physical, emotional, and social factors. Frailty, diminished autonomy, and dependence on caregivers were identified as factors that predispose these individuals to the risk of distress violence and neglect.

*Because they’re more vulnerable, especially physically, I think they’re much more susceptible to violence. They depend on someone else to care for them.* (I1NUT)

*I saw my grandparents gradually losing their independence due to illness, back pain, and so on.* (I2MED)

*In my grandmother’s case,* [...] *she could no longer cook, and we had to help her with everything, like cutting vegetables and preparing meals.* (I3PED)

Respondents also experienced the emotional impact of aging, culminating in depression and social isolation.

*Both my maternal and paternal great-grandmothers were very independent, but as they aged, physical frailty and depression gradually set in.* (I3LAW)

### Considering the trivialization of elder abuse

Participants expressed concern about the normalization and invisibility of elder abuse in society. They identified several contributing factors, including social indifference, normalization, the hidden nature of the abuse, and a lack of reporting mechanisms, which results in the trivialization of the act as a problem that causes discomfort and distress.

*Society tends to think this is more common, trivializing elder abuse somewhat; that’s what I see.* (I2CIVENG)

*Because it is something widely known that happens, but no one talks about it, people act naturally, thinking it is normal, when it is not.* (I3CIVENG)

Additionally, older adults may not identify certain behaviors, such as verbal aggression or neglect, as violence.

*Many older adults do not believe that certain actions constitute violence and often remain silent due to fear or lack of awareness.* (I1NUR)

The COVID-19 pandemic has been identified as a factor that exacerbated elder abuse by increasing social isolation.

*When the pandemic began, I noticed an increase in reports of elder abuse. Isolation made the situation worse for many older adults.* (I1TO)

### Proposing actions to reduce elder abuse

Participants proposed measures to combat elder abuse. They advocated incorporating the topic of elder abuse into undergraduate curricula across all programs, highlighting the need to prepare future professionals to recognize and address the problem effectively. This can be seen in the following statements:

*All professions end up dealing with older adults* [...] *so, I think it would be important in all courses to understand what these people’s lives are like.* (I1MED)

*It’s not just in healthcare. Engineers, for instance, need to consider older adults’ needs when designing spaces.* (I3NUT)

Participants emphasized the need to make public policies effective. Furthermore, they referred to the tendency to limit awareness-raising efforts to commemorative dates, advocating for ongoing and sustainable initiatives.

*I think there are specific policies for older adults’ health, but they need more funding to be effectively implemented.* (I2NUR)

*I see campaigns during Older Adult Day, but after that, everyone forgets.* (I2MED)

Participants also highlighted the importance of fostering a social culture that values older adults, respects them, and encourages their autonomy in the pursuit of healthy aging. They suggested looking to Eastern cultures as a model for how to treat older adults.

[...] *we should encourage more preventive actions* [...]*, improve people’s aging in general, how people grow and age. I think this whole process should be much better* [...]*, control of risk factors, everything else, because, really, as we age, the body itself is more fragile* [...]. (I2NUR)

*So, if she has physical and mental health, she will have better health at home and will be able to have autonomy to do small activities. And I think that, in cases of older adults who are losing autonomy in daily activities, eating, bathing* [...] *I think the family could be encouraged* [...]. (I3MED)

*While I like Western cultures, I admire how Eastern cultures, like Japan, really value their older adults.* (I2LAW)

In conclusion, in the data validation process, participants expressed agreement and satisfaction with the theoretical model constructed, noting that the collected data were addressed comprehensively, and the interpretation effectively reflects university students’ understanding of the phenomenon.

**Figure 1:**
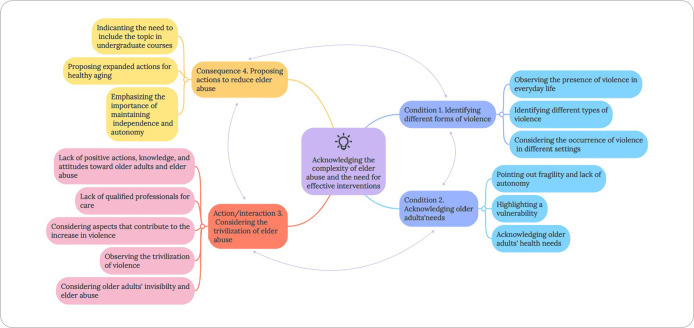
Theoretical model of university students’ experiences, developed in Nvivo software and illustrated in MindMeister software, Marília, São Paulo, Brazil, 2025

After analyzing each component and their respective connections, the theoretical model was developed with the central category identified as “Acknowledging the complexity of elder abuse and the need for effective interventions”, as seen in Figure 1. A condition of the phenomenon is that the university students identified the different types of violence and that it occurs both at home and in institutions. Furthermore, they recognized that older adults’ needs arise from the fragility and vulnerability inherent in the aging process, which contributes to increased violence. In an action/interaction movement, they highlighted the trivialization of elder abuse, as society in general naturalizes it, in addition to the lack of preparedness to address the problem. Consequently, they proposed actions to reduce violence, such as implementing public policies, including the topic in undergraduate curricula, expanding initiatives for healthy aging, and maintaining older adults’ independence and autonomy.

## DISCUSSION

The analysis of interviews with university students from various courses about experiences related to elder abuse motivated the development of the theoretical model “Acknowledging the complexity of elder abuse and the need for effective interventions”.

Initially, it was clear that the interviewees were able to identify the different manifestations of elder abuse. The types of violence discussed can be seen as acts of coercion, and for this phenomenon, which has become more visible in liquid modernity, it is crucial to develop interdisciplinary approaches^(13)^.

Participants reported that elder abuse is recognized through personal experiences, media representations, and stories from those around them. While the technological revolution has facilitated the faster dissemination of information, it also risks normalizing violent acts. The media often inundates the public with images of human distress, which can result in desensitization^(13)^.

Despite acknowledging elder abuse as a significant problem, participants noted that it receives insufficient attention from society, more specifically from healthcare services that encounter difficulties in managing it^(3)^. This observation aligns with the concept of liquid modernity, which highlights the commodification of public life while eroding meaningful interpersonal connections^(13)^.

Elder abuse predominantly occurs in the home and is often perpetrated by family members. In this environment, victims may experience various types of violence, including domestic abuse, modern slavery, and denial of spiritual rights^(12)^. The family, traditionally seen as a source of security, has seen its bonds weakened in liquid modernity, turning older adults who were once respected and valued into burdens, especially when they become dependent on others for daily activities^(16)^.

Regarding violence in family environments, it is important to consider that family caregivers can often become negligent or aggressive towards the older adult, due to physical exhaustion, conflicts with the older adult or due to having a mental disorder, whether due to the use of illicit drugs or not^(17)^. In contrast, family members who recognize the value of their relatives as older adults, and even when overwhelmed, tend to remain committed to caregiving^(18)^.

In NHs, participants reported the existence of neglect. To address this issue, strategic awareness and training for staff in these settings is essential^(19)^. While these strategies are urgent, emphasis must be placed on human relationships, especially with family members, who represent these individuals’ primary source of support. In this case, education can be an opportunity to redefine the structures that guide social actions amid the postmodern crisis^(20)^.

Participants identified frailty in older adults-defined as a decrease in functional and intrinsic capacities-and vulnerabilities, which increase susceptibility to violence and reduce protection in adverse situations. This understanding aligns with research that correlates frailty with elder abuse and depression^(21)^. Therefore, the importance of promoting the autonomy and independence of older adults is highlighted.

Given the growth of the older adult population and its inherent vulnerabilities, it is worrying that postmodernity often promotes neglect, individualism, and indifference to others’ needs. This phenomenon is described as “moral blindness”, a condition marked by the loss of human sensitivity to others’ distress, leading to justifications that, although rationalized, are fundamentally immoral^(12)^.

Rapid social transformations have led to the naturalization of facts, individuals, and memories. Participants recognized the naturalization of elder abuse within the broader social context. In contemporary society, across various cultures, there is often a prevailing indifference to surrounding events, a phenomenon known as the “adiaphorization of human behavior”. This indifference leads individuals to become desensitized to the horrors of war, murder, and conflict, ultimately diminishing their ability to respond to such tragedies^(12)^.

By acknowledging the trivialization of elder abuse, it can be concluded that university students were not entirely indifferent to these issues. They recognized various types of violence, including neglect, omission, abandonment, and restriction, particularly within family relationships. Furthermore, they observed how this violence is often normalized, occurring discreetly and covertly^(12)^.

Regarding the lack of positive actions, knowledge, and attitudes about older adults highlighted by participants, there is an urgent need to restore a sense of morality. This involves promoting a more reflective approach to these issues that affect humanity and cultivating greater commitment to others-beyond mere conventions and social laws-through the acknowledgment of individual worth. To achieve this, it is crucial to restore human sensitivity, actively confronting the fear and indifference that are increasingly prevalent in society. This requires taking responsibility and committing to others’ well-being^(12,13)^.

Interdisciplinary practice has been considered effective in promoting social and intergenerational sustainability, through awareness and engagement, with a view to promoting respect, care and protection for older adults, which can be done through interactive and collaborative educational technologies among students from different areas of knowledge^(8)^.

Furthermore, the interdisciplinary perspective reveals the need to raise awareness among younger generations through educational initiatives and skills training, aiming to improve the approach to case tracking and prevention of elder abuse. Strengthening strategies that integrate social and health policies has also been shown to reduce elder abuse in India^(22)^.

Current research indicates that participants believe that elder abuse often occurs covertly. In this regard, it has been observed that, even in countries like Iran, older adults are culturally respected, and abuse of this population remains a hidden problem. They recommend improving medical and social service programs for prevention, diagnosis, assessment, and intervention^(23)^.

Regarding the influence of culture on the valorization of aging and older adults, it is important to highlight that in Eastern countries such as China, where older adults are traditionally valued, incidents of elder abuse have increased. This increase is attributed to changes in the socioeconomic landscape and the decline in family size^(24)^.

Thus, it is clear that, with the exception of financial abuse, older adults with low levels of social support are more susceptible to all other types of abuse, which emphasizes that strengthening policies aimed at older populations is crucial to prevent all types of abuse and should be treated as a public health priority^(25)^.

From this perspective, when addressing the various types of elder abuse worldwide, this population’s historical devaluation is highlighted. Thus, it is argued that this is an issue that must include: public health, through primary, secondary, and tertiary prevention strategies; medicine, which focuses on early detection; social services, which offer intervention through protective services for vulnerable adults; the legal field, which addresses the issue in forensic centers and in the training of legal professionals; and the human rights sector, which works to increase the visibility of elder abuse at a macro level^(5)^.

The need to incorporate the topic of elder abuse into undergraduate curricula is emphasized on the grounds that training and education on this topic significantly improves case identification, reporting, and general knowledge^(26,27)^. From this perspective, educational policies recognize the importance of including social sustainability in the curriculum. Furthermore, to effectively address elder abuse, reflections on human behavior are necessary and must be integrated into educational processes, ensuring that all types of elder abuse are recognized as cruel and immoral, and that appropriate preventive measures are implemented^(13)^.

Finally, the findings of this research align with the World Health Organization’s^(4)^ guidelines for reducing elder abuse, which advocate the implementation of actions that promote social well-being, education, and health. Echoing Bauman’s^(13)^ insights, they highlight the need for humanity to seek unity in diversity to promote the acknowledgment of others’ dignity.

It also refers to education that promotes self-reflection, reclaims the ontological meaning of human development, embraces the idea of transformation, and aims to raise awareness, inspired by the hope of regaining a restored humanity. Therefore, the goal is to promote a culture of respect and care for older adults^(28)^.

### Study limitations

This study was conducted in a single municipality. Furthermore, the predominance of health students in the sample may have biased perceptions of the phenomenon. Future research could expand the sample to include students from different regions and programs, promoting a more comprehensive understanding.

### Contributions to nursing, health or public policy

The interdisciplinary approach of this study and the resulting theoretical model offer significant contributions to nursing, health sciences, and university education in various fields of scientific knowledge, by raising awareness of the need for intervention strategies that foster more compassionate and preventive practices against elder abuse through the restoration of humane actions. As a discipline that works interdisciplinarily to produce comprehensive care, nursing directly benefits from the developed theoretical model, which, based on Bauman’s framework, highlights the social and moral dimensions of violence. This theoretical basis strengthens professional training and supports educational and preventive actions, promoting a critical and inclusive approach to care for older adults.

## FINAL CONSIDERATIONS

The study achieved its objective by interpreting university students’ experiences with elder abuse and developing a theoretical model that highlights the complexity of the phenomenon. The findings reveal that students recognize multiple types of violence associated with older adults’ fragility and vulnerability, in addition to denouncing the trivialization of the issue in society. From this understanding, proposals for addressing the issue emerge, including professional training, encouraging older adult autonomy, and strengthening public policies.

The interdisciplinary approach adopted constitutes a significant contribution, including students from different fields of knowledge and proposing an analysis that articulates social, moral, and institutional aspects. By aligning empirical data with Bauman’s theoretical framework, the model broadens our understanding of violence in the context of liquid modernity, highlighting the need to restore ethical sensitivity in human relationships, especially with vulnerable populations.

Based on the findings, we recommend reformulating undergraduate curricula to systematically include content on aging and elder abuse, as well as implementing interprofessional educational initiatives and community-focused outreach programs. Such measures can contribute to the development of critical professionals committed to promoting older adults’ dignity and rights.

## Data Availability

The research data are available in a repository: https://figshare.com/s/4ffb65b417a3eeae1033.
