# Supplementary material for: University students’ experiences regarding elder abuse: Grounded Theory
Source: Rev Bras Enferm. 2026 Jan 9;78(Suppl 3):e20250290. doi: 10.1590/0034-7167-2025-0290 (PMC12798863; doi:10.1590/0034-7167-2025-0290)
Supplement: Translation

# Translation: Vivências de universitários com a violência contra a pessoa idosa: Teoria Fundamentada nos Dados

**DOI:** 10.1590/0034-7167-2025-0290pt

**Authors:** Juliana Ribeiro da Silva Vernasque, Maria Clara de Sousa Santos, Ana Laura Lopes Loosli, Larissa Rodrigues, Paula Sales Rodrigues, Fabiana Veronez Martelato Gimenez, Miriam Fernanda Sanches Alarcon, Maria José Sanches Marin

**Affiliations:** I Faculdade de Medicina de Marília. Marília, São Paulo, Brasil; II Universidade Estadual do Norte do Paraná. Bandeirantes, Paraná, Brasil

**Keywords:** Abuso de Idosos, Violência, Universidades, Estudantes, Teoria Fundamentada.

## Abstract

**Objetivos:**

interpretar as vivências de jovens universitários sobre violência contra pessoa idosa e desenvolver um modelo teórico.

**Métodos:**

estudo qualitativo, baseado na abordagem straussiana da Teoria Fundamentada e no referencial teórico de Bauman, com amostragem por saturação teórica, em que foram entrevistados 34 universitários de diversas áreas. O processamento dos dados utilizou o *software* NVivo. O estudo foi aprovado pelo Comitê de Ética em Pesquisa com Seres Humanos.

**Resultados:**

os participantes apontaram para diferentes formas de violência contra as pessoas idosas, bem como invisibilidade e banalização na sociedade. Identificaram-se a fragilidade e vulnerabilidade das pessoas idosas como fatores de risco, sugerindo-se estratégias preventivas, além da inclusão do tema nos currículos acadêmicos, promoção do envelhecimento saudável e fortalecimento de políticas públicas.

**Considerações Finais:**

o modelo teórico desenvolvido aponta para necessidade urgente de intervenções educativas pautadas na compreensão crítica desse problema, com ênfase na sensibilidade humana e na moralidade das interações sociais.

## INTRODUÇÃO

A atual transição demográfica e epidemiológica, resultante do envelhecimento populacional, exige esforços sociais significativos, pois as pessoas idosas têm necessidades complexas e crônicas, as quais incluem o gerenciamento de condições de saúde de longo prazo, a garantia de acesso a cuidados de saúde adequados, e o enfrentamento de desafios sociais, emocionais e econômicos associados ao envelhecimento. Essa questão é particularmente urgente em países menos desenvolvidos, em que o ritmo rápido do envelhecimento populacional exacerba as inadequações existentes, como infraestrutura de saúde e políticas insuficientes para apoiar as pessoas idosas, além da falta de conscientização pública sobre a complexidade do envelhecimento^(1)^.

O processo de envelhecimento envolve mudanças morfológicas, funcionais, bioquímicas e psicológicas^(2)^ que podem resultar em perda da capacidade funcional, surgimento de doenças, síndromes geriátricas, hospitalizações, e conflitos sociais, familiares e intergeracionais. Consequentemente, as pessoas idosas experimentam perda de autonomia e independência, aumentando a exposição a fatores de risco, incluindo a violência^(3)^.

O crescimento global da população com mais de 60 anos tem resultado em alta prevalência de abuso contra as pessoas idosas, evidenciando a necessidade de novas estratégias para enfrentar essa questão. Essa violação dos direitos humanos envolve qualquer ação ou omissão prejudicial em relacionamento de confiança^(4)^.

O abuso contra pessoas idosas é um fenômeno global complexo e evitável que pode gerar sofrimento psicológico ou físico, privação de liberdade ou até mesmo a morte. Ele se manifesta de várias formas, incluindo negligência, violência autoinfligida, abuso físico, violência sexual, exploração financeira, abandono e abuso psicológico ou emocional. Representa atualmente um problema significativo para a sociedade, principalmente para a saúde pública, demandando ações intersetoriais. No entanto, muitos profissionais relatam limitações na capacidade de intervenção, juntamente com medos de denúncia e incertezas sobre os papéis de outros serviços, complicando ainda mais a resposta aos abusos contra pessoas idosas^(3)^.

Como questão interdisciplinar, o abuso de pessoas idosas envolve as ciências humanas, exatas e biológicas. No entanto, muitas vezes, é subfinanciado, subexaminado e subreconhecido, recebendo atenção inadequada de profissionais em todos os campos^(5)^.

Diante desse contexto, a formação de futuros profissionais, de todas as áreas do conhecimento, pode desempenhar papel fundamental na identificação e prevenção de abusos contra pessoas idosas, preparando universitários para se tornarem agentes de mudança^(6)^.

As universidades, por meio de seu papel de ensino, pesquisa e prestação de serviço à comunidade, desempenham a relevante função de difusão de valores e crenças que levam ao desenvolvimento de ações cotidianas que podem influenciar positivamente a visão de mundo da sociedade, criando uma cultura voltada aos preceitos de humanização, especialmente quando se trata de uma sociedade em franco processo de envelhecimento e com demandas específicas^(7)^.

Acrescenta-se que as universidades podem conduzir a formação de profissionais conscientes e com visão abrangente de sustentabilidade, economia inclusiva e justiça social, sendo que esta abordagem deve ser integrada ao currículo escolar de forma transversal em todas as áreas do conhecimento, por meio de práticas pedagógicas ativas que visam à reflexão crítica sobre a realidade e sua transformação^(8)^.

Compreende-se, assim, que estudantes universitários representam segmento de futuros profissionais que se envolverão diretamente com populações mais velhas e se constituem importantes agentes de mudanças nas suas distintas inserções. No entanto, estudos indicam que o tópico referente a abuso de pessoas idosas raramente é abordado sistematicamente nos currículos de graduação, limitando a preparação desses futuros profissionais para intervir em situações de violência^(9)^.

Diante desse contexto, surgiu a questão: como estudantes universitários de diferentes áreas de estudo vivenciam a violência contra pessoas idosas?

## OBJETIVOS

Interpretar as vivências de jovens universitários sobre a violência contra pessoa idosa e desenvolver um modelo teórico.

## MÉTODOS

### Aspectos éticos

A pesquisa obteve aprovação do Comitê de Ética em Pesquisa, seguindo os preceitos éticos do Conselho Nacional de Saúde. As entrevistas foram realizadas utilizando o *Google Meet* ^®^, e os participantes assinaram o Termo de Consentimento Livre e Esclarecido, após compreensão e anuência. Para manter o anonimato, os entrevistados foram identificados pela letra “E”, seguida do número da ordem da entrevista e das iniciais da área de formação, como ciências sociais (CIESOC), direito (DIR), enfermagem (ENF), engenharia civil (ENGCIV), engenharia elétrica (ENGELE), medicina (MED), nutrição (NUT), pedagogia (PED) e terapia ocupacional (TO).

### Tipo de estudo

Trata-se de pesquisa qualitativa que utilizou a Teoria Fundamentada nos Dados (TFD), na vertente straussiana, a qual é construída por meio da interação entre o pesquisador e o próprio dado^(10)^. A abordagem straussiana foi escolhida pela capacidade de explorar processos sociais dinâmicos e desvendar as interações complexas entre fatores sociais, culturais e institucionais subjacentes ao abuso de pessoas idosas. Esta abordagem metodológica permite maior envolvimento do pesquisador na interpretação e modelagem de teorias emergentes, garantindo compreensão abrangente do fenômeno^(10)^.

Para manter o rigor metodológico, utilizou-se o *protocolo COnsolidated criteria for REporting Qualitative research* como ferramenta de apoio para o desenvolvimento da pesquisa.

### Referencial teórico

O modelo teórico é baseado no trabalho de Zygmunt Bauman, que reconhece a violência como parte de um processo complexo na humanidade. Bauman postula que, sem intervenção para interromper os ciclos de violência, as vítimas de hoje podem se tornar os agressores de amanhã^(11-13)^.

Ademais, a sociedade contemporânea é caracterizada pela “liquidez” das relações sociais, em que a fragilidade das redes de apoio e a inconsistência nas interações interpessoais afetam diretamente a vulnerabilidade dos indivíduos, particularmente das pessoas idosas^(11)^. Essa fragilidade das relações humanas e sociais contribui para marginalização das pessoas idosas e perpetuação do abuso, apresentando desafio significativo para as futuras gerações de profissionais.

### Cenário do estudo

O estudo foi realizado em município de médio porte da região Centro-Oeste do estado de São Paulo, com população estimada de 237.627 habitantes para 2022, sendo 44.835 com 60 anos ou mais^(14)^. O município concentra amplo complexo de Instituições de Ensino Superior (IES), englobando tanto instituições públicas quanto privadas que oferecem cursos em diversas áreas do conhecimento. Para este estudo, selecionaram-se, por conveniência, quatro IES, sendo duas públicas e duas privadas, com objetivo de incluir universitários das áreas das ciências da saúde, exatas e humanas para uma composição amostral diversificada, conforme sugerido pela TFD, e obter variação teórica e profundidade analítica.

### Amostra

A amostra foi definida por saturação teórica, sendo que a mesma foi considerada plenamente alcançada na trigésima quarta entrevista, realizada com estudantes universitários das áreas das humanidades, ciências exatas e ciências da saúde oriundos de duas instituições públicas e duas privadas. Os critérios de inclusão foram ser aluno matriculado em uma das quatro instituições participantes, ter completado pelo menos um ano de estudo e ter 18 anos ou mais. Os critérios de exclusão foram não ter disponibilidade para participar da entrevista ou ser estudante transferido de outra instituição há menos de seis meses.

### Coleta dos dados

Entrevistas semiestruturadas serviram como o principal método de coleta de dados, apropriado para investigar fenômenos sociais e capturar percepções individuais^(15)^_._ As entrevistas, conduzidas por uma doutoranda com experiência em técnicas de entrevista, ocorreram de janeiro de 2022 a setembro de 2023. Esse período permitiu que cada entrevista fosse transcrita e analisada sequencialmente em um processo contínuo.

Ao utilizar o roteiro, coletaram-se informações sociodemográficas (idade, sexo, estado civil, ano de estudo e área de estudo), incluindo as seguintes perguntas orientadoras: o que você sabe sobre violência contra pessoas idosas que pode compartilhar conosco? Como você percebe a violência contra pessoas idosas na sociedade? Qual é a importância de abordar este tópico durante a graduação? Você tem sugestões para melhorar a abordagem sobre este assunto? O que este tópico significa para você pessoalmente? Como você responderia a uma situação envolvendo abuso de pessoas idosas?

Cada entrevista durou, aproximadamente, 30 minutos, e foi transcrita e analisada imediatamente depois. Foram conduzidas de forma focada no objeto de estudo, e a transcrição e análise concomitante permitiram pequenos ajustes nas perguntas, conforme necessário, garantindo compreensão aprofundada do tópico estudado.

### Análise dos dados

A análise foi realizada conforme os princípios da TFD, na vertente straussiana, envolvendo as etapas de “codificação aberta”, “codificação axial” e “codificação seletiva”^(15)^. O *software* NVivo 14 foi utilizado para facilitar a organização e sistematização dos dados qualitativos, garantindo rastreabilidade, consistência e fidedignidade do processo analítico. A codificação foi realizada pelo primeiro autor e verificada pelo último autor, para garantir precisão e confiabilidade. O *software MindMeister* foi utilizado para ilustração do modelo teórico.

O processo de codificação seguiu caminho contínuo, conceituando dados de acordo com as características, comparando e agrupando conceitos, o que gerou categorias preliminares e, finalmente, a categoria central.

Inicialmente, os dados foram submetidos à microanálise linha por linha para identificar códigos significativos derivados diretamente das transcrições das entrevistas. Esta etapa resultou em 577 códigos. Por exemplo, ao relatar a leitura de um conto de Clarice Lispector sobre abandono de uma idosa, um estudante afirmou: *“Quando fala em violência contra idoso, a primeira coisa que me vem é um conto da Clarice* [...] *sempre impactou muito, uma cena que ficou é ela toda suja de migalhas de pão”.* A partir desse trecho, foram codificadas ações como “nomeando o conto”, “relacionando com abandono” e “expressando impacto emocional”, evidenciando como narrativas literárias influenciam a percepção sobre violência e abandono de pessoas idosas.

Posteriormente, na codificação aberta, os códigos foram agrupados em conceitos ou elementos que compartilhavam similaridades temáticas, produzindo 50 conceitos. Por exemplo, os códigos *“Compreendendo a negligência como uma violência contra a pessoa idosa”* (E1ENGCIV) e *“Considerando que a população idosa é negligenciada e que a violência ocorre muito em âmbito familiar e nas instituições”* (E2ENF) foram reunidos sob o conceito/elemento “negligência”.

A codificação axial envolveu o estabelecimento de relações entre os 50 conceitos/elementos para formulação das 14 categorias preliminares/subcategorias, possibilitando uma compreensão mais profunda da dinâmica subjacente ao abuso de pessoas idosas a partir das vivências dos universitários.

A etapa final, codificação seletiva, foi refinada em quatro categorias, integrando as 14 subcategorias com base em propriedades e dimensões compartilhadas para formar a categoria central, o que permitiu sintetizar os processos interconectados das vivências dos estudantes relacionadas ao abuso de pessoas idosas.

A validade do modelo foi fortalecida por meio de revisões em duplas por pesquisadores experientes na TFD e pela validação externa em dois encontros remotos, que contaram com a participação de cinco estudantes entrevistados, dois especialistas em TFD e três membros do grupo de pesquisa, confirmando a representatividade e a fidelidade interpretativa do modelo teórico proposto.

## RESULTADOS

Participaram do estudo 34 estudantes universitários, oriundos de diferentes áreas do conhecimento, cursos e IES. Entre os participantes, 22 se identificaram como mulheres (64,7%), e 12, como homens (35,3%). A idade variou entre 19 e 44 anos, com média de 24,47 anos, revelando um grupo predominantemente jovem, porém com faixas etárias variadas.

A composição da amostra contemplou estudantes de nove cursos distintos, distribuídos entre os 2º e 5º anos da graduação, conforme detalhado no Quadro 1. Essa diversidade contribuiu para ampliar o alcance das percepções e reflexões sobre a temática investigada. No que se refere à instituição de origem, 18 participantes estavam matriculados em universidades públicas e 16 em instituições privadas, reforçando o compromisso do estudo com a representatividade institucional.

**Quadro 1:** Características dos participantes segundo curso e ano de graduação, Marília, São Paulo, Brasil, 2025

Curso/ano de graduação	CIE SOC	DIR	ENF	ENG CIV	ENG ELE	MED	NUT	PED	TO	Total geral
2º ano	2	4		1				2		9
3º ano	1			1	1		3	1	1	8
4º ano			4	1		4	1		3	13
5º ano				4						4
**Total geral**	**3**	**4**	**4**	**7**	**1**	**4**	**4**	**3**	**4**	**34**

*CIESOC - ciências sociais; DIR - direito; ENF - enfermagem; ENGCIV - engenharia civil; ENGELE - engenharia elétrica; MED - medicina; NUT - nutrição; PED - pedagogia; TO - terapia ocupacional.*

O processo de codificação revelou como fenômeno central “Reconhecendo a complexidade da violência contra a pessoa idosa e necessidade de intervenções efetivas”, que foi evidenciado pelas categorias “Identificando as diferentes formas de violência”, “Reconhecendo as necessidades das pessoas idosas”, “Considerando a banalização da violência contra as pessoas idosas” e “Propondo ações para reduzir o abuso de pessoas idosas”, conforme se apresenta a seguir.

### Identificando diferentes formas de violência

Nesta categoria, os entrevistados reconheceram as diferentes formas de violência, que ela ocorre tanto no domicílio quanto em instituições, perpetrada principalmente por familiares.

Para os participantes, as formas como a violência contra pessoas idosas se manifestam incluem violência física, violência financeira, violência psicológica, violência sexual, negligência, omissão, desrespeito, idadismo e negação da dignidade, o que se traduz em atos de privação, abandono, isolamento, impaciência e insultos, conforme se observam nas falas que seguem.

*Porque não é só violência física. O que a gente vê é abuso financeiro, que as pessoas idosas sofrem de familiares que se aproveitam deles, abuso emocional, violência psicológica e sexual também* [...]. (E2TO)

*A violência contra as pessoas idosas, muitas vezes, não é evidente. Pode se manifestar na falha em ajudar um idoso.* (E4DIR)

*Interferir na dignidade deles, querer menosprezá-los por causa da idade, não dar a atenção que eles precisam.* (E4NUT)

Essas formas de violência, geralmente, ocorrem em ambientes domésticos, principalmente perpetradas por familiares. Além disso, os participantes destacaram que ambientes institucionais, como serviços de saúde e Instituições de Longa Permanência de Idosos (ILPIs), também apresentam riscos de negligência, abandono e isolamento prolongado.

*Havia uma senhora idosa que morava sozinha e era maltratada pelos filhos. Eles extorquiam todo o dinheiro que ela recebia da pensão e só a visitavam para cobrar o dinheiro, negligenciando suas necessidades básicas.* (E1MED)

*Na minha cidade, há uma casa de repouso com muitas pessoas idosas que não recebem visitas. A maioria deles parece visivelmente triste, devido à falta da presença da família.* (E4DIR)

Os universitários mostraram ter consciência da existência da problemática que a violência contra a pessoa idosa representa, uma vez que já vivenciaram esse tipo de situação com familiares ou pessoas próximas, souberam por meio da mídia, ou ouviram de outras pessoas.

[...] *ah, eu tenho experiências pessoais, da família e experiências que eu vivi no meu curso, assim.* (E4MED)

[...] *já ouvi casos, grande parte na mídia, que é bastante prevalente essa questão de violência contra idosos.* (E4ENF)

### Reconhecendo as necessidades das pessoas idosas

Os participantes enfatizaram necessidades específicas das pessoas idosas, que ocorrem devido a fatores físicos, emocionais e sociais, sendo que fragilidade, autonomia diminuída e dependência de cuidadores foram identificados como fatores que predispõem essas pessoas ao risco de sofrer violência e negligência.

*Por serem pessoas mais vulneráveis, especialmente em termos de questões físicas, acho que são muito mais suscetíveis à violência. Eles dependem de outra pessoa para cuidar deles.* (E1NUT)

*Vi meus avós perdendo a autonomia aos poucos por causa de doenças, dores nas costas e assim por diante.* (E2MED)

*No caso da minha avó,* [...] *ela não conseguia mais cozinhar, e nós tínhamos que ajudá-la com tudo, como cortar vegetais e preparar refeições.* (E3PED)

Os entrevistados também vivenciaram o impacto emocional causado pelo envelhecimento, culminando em depressão e isolamento social.

*Tanto minhas bisavós maternas quanto paternas eram muito independentes, mas à medida que envelheciam, a fragilidade física e a depressão gradualmente se instalaram.* (E3DIR)

### Considerando a banalização da violência contra as pessoas idosas

Os participantes expressaram preocupação com a normalização e invisibilidade do abuso de pessoas idosas na sociedade. Eles identificaram vários fatores contribuintes, incluindo indiferença social, normalização, natureza oculta do abuso e carência de mecanismos de denúncia, o que resulta em banalização do ato enquanto problema que causa mal-estar e sofrimento.

*A sociedade tende a achar isso mais comum, banalizando um pouco o abuso de pessoas idosas; é isso que vejo.* (E2ENGCIV)

*Por ser algo amplamente conhecido que acontece, mas ninguém fala sobre isso, as pessoas agem naturalmente, pensando que é normal, quando não é.* (E3ENGCIV)

Além disso, as pessoas idosas podem não identificar certos comportamentos, como agressão verbal ou negligência, como violência.

*Muitas pessoas idosas não acreditam que certas ações sejam violência e, frequentemente, permanecem em silêncio, devido ao medo ou à falta de consciência.* (E1ENF)

A pandemia de COVID-19 foi identificada como fator que exacerbou a violência contra pessoas idosas, ao aumentar o isolamento social.

*Quando a pandemia começou, notei um aumento nos relatos de abuso de pessoas idosas. O isolamento piorou a situação para muitas pessoas idosas.* (E1TO)

### Propondo ações para reduzir o abuso de pessoas idosas

Os participantes propuseram medidas para combater o abuso de pessoas idosas. Eles defenderam a incorporação do tópico de abuso de pessoas idosas nos currículos de graduação em todos os cursos, destacando a necessidade de preparar futuros profissionais para reconhecer e abordar o problema de forma eficaz. Isso pode ser observado nas falas a seguir:

*Todas as profissões acabam lidando com pessoas idosas* [...] *então, acho que seria importante em todos os cursos entender como é a vida dessas pessoas.* (E1MED)

*Não é apenas na área da saúde. Engenheiros, por exemplo, precisam considerar as necessidades das pessoas idosas ao projetar espaços.* (E3NUT)

Os participantes enfatizaram a necessidade de tornar efetiva as políticas públicas. Além disso, fazem referência à tendência de confinar os esforços de conscientização a datas comemorativas, defendendo iniciativas contínuas e sustentáveis.

*Acho que há políticas específicas para a saúde das pessoas idosas, mas elas precisam de mais financiamento para serem efetivamente implementadas.* (E2ENF)

*Vejo campanhas durante o Dia do Idoso, mas, depois disso, todo mundo esquece.* (E2MED)

Os participantes destacaram, ainda, a importância de promover cultura social que valorize as pessoas idosas, respeite e incentive a autonomia delas, na busca de um envelhecimento saudável. Eles sugeriram olhar para as culturas orientais como um modelo de como tratar as pessoas idosas.

[...] *a gente deveria incentivar mais ações preventivas* [...], *melhorar o envelhecimento em geral das pessoas, como as pessoas crescem e envelhecem. Eu acho que esse processo todo tinha que ser muito melhor* [...], *controle de fatores de risco, tudo mais, porque, realmente, num envelhecimento, o corpo em si ele é mais fragilizado* [...]. (E2ENF)

*Então, ela tendo uma saúde física, uma saúde mental, ela vai ter uma saúde melhor dentro de casa e vai conseguir ter autonomia para fazer pequenas atividades. E eu acho que, em casos de idosos que vão perdendo de fato autonomia para atividades cotidianas, para comer, para tomar banho* [...] *acho que poderia ser estimulada a família* [...]. (E3MED)

*Embora eu goste das culturas ocidentais, admiro como as culturas orientais, como o Japão, realmente valorizam suas pessoas idosas.* (E2DIR)

Concluindo, no processo de validação dos dados, os participantes expressaram concordância e satisfação com o modelo teórico construído, observando que os dados coletados foram abordados de forma abrangente, e a interpretação reflete efetivamente a compreensão do fenômeno pelos estudantes universitários.

Após análise de cada componente e das respectivas conexões, o modelo teórico foi desenvolvido com a categoria central identificada como “Reconhecendo a complexidade da violência contra a pessoa idosa e necessidade de intervenções efetivas”, como se observa na Figura 1. Revela-se como condição do fenômeno que os universitários identificaram as diferentes formas de violência e que ela ocorre tanto no domicílio quanto em instituições. Além disso, eles reconhecerem que as necessidades da pessoa idosa são decorrentes da fragilidade e vulnerabilidade presentes no processo de envelhecimento, o que contribui para aumentar a violência. Em um movimento de ação/interação, pontuaram banalização da violência contra a pessoa idosa, uma vez que a sociedade em geral naturaliza esse fato, além do despreparo para o enfrentamento do problema. Como consequência, fizeram proposições de ações para diminuir a violência, como efetivação das políticas públicas, inclusão da temática nos currículos dos cursos de graduação, ampliação das ações para um envelhecimento saudável, e manutenção da independência e autonomia do idoso.

**Figura 1:** Modelo teórico das vivências dos estudantes universitários, elaborado no *software* **Nvivo e ilustração no** *software MindMeister*, Marília, São Paulo, Brasil, 2025

## DISCUSSÃO

A análise de entrevistas com estudantes universitários de diversos cursos sobre vivências relacionadas à violência contra pessoas idosas motivou o desenvolvimento do modelo teórico “Reconhecendo a complexidade da violência contra a pessoa idosa e necessidade de intervenções efetivas”.

Inicialmente, ficou evidente que os entrevistados conseguiam identificar as diferentes manifestações de abuso de pessoas idosas. Os tipos de violência discutidos podem ser vistos como atos de coerção e, para esse fenômeno, que se tornou mais visível na modernidade líquida, é crucial criar abordagens interdisciplinares^(13)^.

Os participantes relataram que a violência contra pessoas idosas é reconhecida por meio de experiências pessoais, representações na mídia e histórias daqueles ao redor. Embora a revolução tecnológica tenha facilitado a disseminação mais rápida de informações, ela também corre o risco de normalizar atos violentos. A mídia, frequentemente, inunda o público com imagens de sofrimento humano, o que pode resultar em dessensibilização^(13)^.

Apesar de reconhecer o abuso de pessoas idosas como problema significativo, os participantes notaram que este recebe atenção insuficiente da sociedade, mais especificamente dos serviços de saúde que encontram dificuldades no manejo^(3)^. Essa observação se alinha com o conceito de modernidade líquida, que destaca a mercantilização da vida pública, enquanto corrói conexões interpessoais significativas^(13)^.

A violência contra pessoas idosas ocorre predominantemente em casa, sendo, muitas vezes, perpetrado por familiares. Nesse ambiente, as vítimas podem sofrer vários tipos de violência, incluindo abuso doméstico, escravidão moderna e negação de direitos espirituais^(12)^. A família, tradicionalmente vista como fonte de segurança, viu os laços enfraquecidos na modernidade líquida, tornando pessoas idosas que antes eram respeitadas e valorizadas em fardos, principalmente quando se tornam dependentes de outros para atividades diárias^(16)^.

Em relação à violência no ambiente familiar, é importante considerar que os cuidadores familiares, muitas vezes, podem se tornar negligentes ou agressivos com a pessoa idosa, devido à exaustão física, conflitos com o idoso ou por ser portador de transtorno mental, seja pelo uso de drogas ilícitas ou não^(17)^. Em contraste, os familiares que reconhecem o valor de seus parentes enquanto pessoas idosas, e mesmo quando sobrecarregados, tendem a permanecer comprometidos com os cuidados^(18)^.

Em ILPIs, os participantes indicaram existência de negligência. Para abordar essa questão, a conscientização estratégica e o treinamento para a equipe nesses ambientes são essenciais^(19)^. Embora essas estratégias sejam urgentes, é necessária ênfase nos relacionamentos humanos, principalmente quanto aos familiares que representam a principal fonte de apoio para essas pessoas. Neste caso, a educação pode ser oportunidade de redefinir as estruturas que orientam as ações sociais em meio à crise pós-moderna^(20)^.

Os participantes identificaram fragilidade em pessoas idosas - definida como diminuição nas capacidades funcionais e intrínsecas - e vulnerabilidades, que aumentam a suscetibilidade à violência e reduzem a proteção em situações adversas. Esse entendimento se alinha com pesquisas que correlacionam fragilidade com abuso de pessoas idosas e depressão^(21)^. Destaca-se, assim, a importância da promoção da autonomia e independência das pessoas idosas.

Diante do crescimento da população idosa e das vulnerabilidades inerentes, é preocupante que a pós-modernidade frequentemente promova negligência, individualismo e indiferença em relação às necessidades dos outros. Esse fenômeno é descrito como “cegueira moral”, condição marcada pela perda da sensibilidade humana ao sofrimento dos outros, levando a justificativas que, embora racionalizadas, são fundamentalmente imorais^(12)^.

As rápidas transformações sociais motivaram a naturalização de fatos, indivíduos e memórias. Os participantes reconheceram a naturalização da violência contra a pessoa idosa dentro do contexto social mais amplo. Na sociedade contemporânea, em várias culturas, muitas vezes, há indiferença predominante aos eventos circundantes, fenômeno conhecido como “adiaforização do comportamento humano”. Essa indiferença leva os indivíduos a se tornarem insensíveis aos horrores de guerras, assassinatos e conflitos, diminuindo, em última análise, a capacidade de responder a tais tragédias^(12)^.

Ao reconhecer a banalização da violência contra pessoas idosas, pode-se concluir que os estudantes universitários não foram totalmente indiferentes a essas questões. Eles reconheceram várias formas de violência, incluindo negligência, omissão, abandono e restrições, particularmente dentro das relações familiares. Além disso, eles observaram como essa violência é frequentemente normalizada, ocorrendo discreta e encobertamente^(12)^.

Em relação à escassez de ações, conhecimento e atitudes positivas sobre pessoas idosas destacadas pelos participantes, há necessidade urgente de restabelecer um senso de moralidade. Isso envolve promover abordagem mais reflexiva a essas questões que afetam a humanidade e cultivar maior comprometimento com os outros - além de meras convenções e leis sociais - por meio do reconhecimento do valor individual. Para atingir isso, é crucial restaurar a sensibilidade humana, confrontando ativamente o medo e a indiferença, cada vez mais prevalentes na sociedade. Isso requer assumir responsabilidade e se comprometer com o bem-estar dos outros^(12,13)^.

A prática interdisciplinar tem sido considerada eficaz na promoção da sustentabilidade social e intergeracional, por meio da conscientização e engajamento, com vistas à promoção do respeito, cuidado e proteção à pessoa idosa, o que pode ser feito por meio de tecnologias educacionais interativas e colaborativas entre estudantes de diferentes áreas do conhecimento^(8)^.

Além disso, a perspectiva interdisciplinar revela a necessidade de aumentar a conscientização entre as gerações mais jovens, por meio de iniciativas educacionais e treinamento de habilidades, visando melhorar a abordagem ao rastreamento de casos e prevenção de abuso de pessoas idosas. O fortalecimento de estratégias que integram políticas sociais e de saúde também demonstrou reduzir o abuso de pessoas idosas na Índia^(22)^.

A pesquisa atual indica que os participantes acreditam que a violência contra pessoas idosas, geralmente, ocorre de forma oculta. Em relação a isso, foi observado, mesmo em países como o Irã, que as pessoas idosas são culturalmente respeitadas, e o abuso dessa população continua sendo um problema encoberto. Eles recomendam o aprimoramento de programas de serviços médicos e sociais para prevenção, diagnóstico, avaliação e intervenção^(23)^.

Em relação à influência da cultura na valorização do envelhecimento e das pessoas idosas, é importante destacar que, em países orientais como a China, em que as pessoas idosas são tradicionalmente valorizadas, os incidentes de violência contra pessoas idosas aumentaram. Esse aumento é atribuído a mudanças no cenário socioeconômico e ao declínio do tamanho da família^(24)^.

Assim, tem-se a constatação de que, com exceção do abuso financeiro, as pessoas idosas com baixos níveis de apoio social são mais suscetíveis a todas as outras formas de abuso, o que enfatiza que o fortalecimento de políticas direcionadas a populações mais velhas é crucial para prevenir todos os tipos de abuso, devendo ser tratado como prioridade de saúde pública^(25)^.

Nessa perspectiva, na abordagem sobre as diversas formas de violência contra pessoas idosas no mundo, é destacada a desvalorização histórica dessa população. Discute-se, assim, que se trata de uma questão que deve incluir: a saúde pública, por meio de estratégias de prevenção primária, secundária e terciária; a medicina, que se concentra na detecção precoce; os serviços sociais, que oferecem intervenção, por meio de serviços de proteção para adultos vulneráveis; o campo jurídico, que aborda a questão em centros forenses e na formação de profissionais do direito; e o setor de direitos humanos, que trabalha para intensificar a visibilidade da violência contra idosos em um nível macro^(5)^.

A necessidade de incorporar o tópico da violência contra pessoas idosas nos currículos de graduação é enfatizada com o argumento de que o treinamento e a educação sobre esse assunto melhoram significativamente a identificação de casos, os relatórios e o conhecimento geral^(26,27)^. Nessa perspectiva, as políticas educacionais reconhecem a importância de inclusão da sustentabilidade social no currículo. Além disso, para abordar eficazmente o abuso de pessoas idosas, são necessárias reflexões sobre o comportamento humano que devem ser integradas aos processos de formação, garantindo que todas as formas de violência contra pessoas idosas sejam reconhecidas como cruéis e imorais, e que medidas preventivas adequadas sejam implementadas^(13)^.

Finalmente, as descobertas desta pesquisa se alinham com as diretrizes da Organização Mundial da Saúde^(4)^ voltadas para redução do abuso de pessoas idosas, que defendem a implementação de ações que promovam o bem-estar social, a educação e a saúde. Ecoando os *insights* de Bauman^(13)^, eles ressaltam a necessidade de a humanidade buscar a unidade na diversidade para promover o reconhecimento da dignidade dos outros.

Remete-se também à educação que promove a autorreflexão, retoma a formação, no sentido ontológico do ser humano, envolve a ideia de transformação e visa à conscientização, inspirada na esperança de se revestir de uma humanidade restaurada. Projeta-se, portanto, a promoção de uma cultura de respeito e cuidado com as pessoas idosas^(28)^.

### Limitações do estudo

Este estudo foi conduzido em um único município. Além disso, a predominância de estudantes da área da saúde na amostra pode ter enviesado a percepção do fenômeno. Pesquisas futuras podem expandir a amostra para incluir estudantes de diferentes regiões e cursos, promovendo compreensão mais abrangente.

### Contribuições para a área da enfermagem, saúde ou política pública

A abordagem interdisciplinar deste estudo e o modelo teórico resultante oferecem contribuições significativas para a enfermagem, as ciências da saúde e a formação universitária em diferentes áreas do conhecimento científico, ao conscientizar sobre a necessidade de estratégias de intervenção que fomentem práticas mais compassivas e preventivas contra o abuso de pessoas idosas, por meio do resgate de ações humanizadas. Como disciplina que atua de forma interdisciplinar para produção da integralidade do cuidado, a enfermagem se beneficia diretamente do modelo teórico desenvolvido, que ao ser fundamentado na estrutura de Bauman, aponta para as dimensões sociais e morais da violência. Essa base teórica fortalece a formação profissional e subsidia ações educativas e preventivas, promovendo abordagem crítica e inclusiva na assistência às pessoas idosas.

## CONSIDERAÇÕES FINAIS

O estudo atingiu seu objetivo ao interpretar as vivências de universitários sobre a violência contra pessoas idosas e desenvolver um modelo teórico que evidencia a complexidade do fenômeno. Os achados revelam que os estudantes reconhecem múltiplas formas de violência associadas à fragilidade e vulnerabilidade da pessoa idosa, além de denunciarem a banalização do tema na sociedade. A partir dessa compreensão, emergem propostas de enfrentamento que envolvem qualificação profissional, incentivo à autonomia da pessoa idosa e fortalecimento das políticas públicas.

A abordagem interdisciplinar adotada constitui uma contribuição relevante, ao incluir estudantes de diferentes áreas do conhecimento e propor uma análise que articula aspectos sociais, morais e institucionais. Ao alinhar os dados empíricos ao referencial teórico de Bauman, o modelo amplia a compreensão sobre a violência no contexto da modernidade líquida, apontando à necessidade de resgate da sensibilidade ética nas relações humanas, especialmente com populações vulneráveis.

Recomendam-se, com base nos achados, a reformulação dos currículos de graduação para inclusão sistemática de conteúdos sobre envelhecimento e violência contra a pessoa idosa, bem como a implementação de ações educativas interprofissionais e programas de extensão voltados à comunidade. Tais medidas podem contribuir para formação de profissionais críticos e comprometidos com promoção da dignidade e dos direitos das pessoas idosas.

## Data Availability

Os dados de pesquisa estão disponíveis em repositório: https://figshare.com/s/4ffb65b417a3eeae1033.
